# Adenocarcinoma among Patients Diagnosed with Lung Cancer in a Tertiary Care Centre: A Descriptive Cross-sectional Study

**DOI:** 10.31729/jnma.7330

**Published:** 2022-04-30

**Authors:** Farmud Ansari, Ram Hari Ghimire, Nischal Shrestha, Sushil Yadav

**Affiliations:** 1 Department of Pulmonary Medicine, Critical Care and Sleep Medicine, Nobel Medical College Teaching Hospital, Kanchanbari, Biratnagar, Nepal; 2 Department of Internal Medicine, Nobel Medical College Teaching Hospital, Kanchanbari, Biratnagar, Nepal

**Keywords:** *adenocarcinoma*, *lung cancer*, *Nepal*, *smoking*

## Abstract

**Introduction:**

Lung cancer is the leading cause of cancer deaths all over the world and adenocarcinoma is the most common type. Diagnosis is made usually at an advanced stage of lung cancer in patients, making it nearly impossible to cure. The aim of this study is to find out prevalence of adenocarcinoma among patients diagnosed with lung cancer in a tertiary care centre.

**Methods:**

A descriptive cross-sectional study was carried out in the Department of Pulmonary Medicine of a tertiary care centre among 69 patients from October, 2018 to September, 2019. Ethical approval was taken from the Institutional Review Committee of a tertiary care hospital (Reference number: 54/2018). A convenience sampling technique was used. Data were entered and analysed using the Statistical Package for the Social Sciences version 21.0. Point estimate at 90% Confidence Interval and descriptive statistics were interpreted as frequency, percentage, or as mean and standard deviations.

**Results:**

Among 69 lung cancer patients, adenocarcinoma was seen in 27 (39.13%) (29.47-48.79 at 90% Confidence Interval). Out of 27, 10 (37.04%) were male and 17 (62.96%) were female. Chronic obstructive pulmonary disease was the major comorbidity seen among 17 (62.96%) patients.

**Conclusions:**

The prevalence of adenocarcinoma was similar to other studies done in similar settings.

## INTRODUCTION

Adenocarcinoma of the lung is the most common type of primary lung cancer, a leading cause of cancer deaths all over the world resulting in 1,761,007 deaths in 2018 (18.4% of all cancer deaths).^1^ Histologically, adenocarcinoma falls under the non-small cell lung cancers type. Lung adenocarcinoma invades mainly the upper region of the lungs.^2^ It is usually diagnosed at a late stage after distant metastases, so the 5-year survival rate is only 7%.^3^

The incidence of adenocarcinoma is increasing in young females and Asian populations.^4^ The burden of adenocarcinoma is rising and timely diagnosis and management are essential for good outcomes.

The aim of this study is to find out prevalence of adenocarcinoma among patients diagnosed with lung cancer in a tertiary care centre.

## METHODS

A descriptive cross-sectional study was carried out in the Department of Pulmonary Medicine, Nobel Medical College Teaching Hospital, Biratnagar, Nepal from October, 2018 to September, 2019 after taking ethical clearance from the Institutional Review Committee (Reference number: 54/2018). Written informed consent was obtained from all the patients.

A convenience sampling technique was used and sample size calculated according to the following formula:

n = (Z^2^ × p × q) / e^2^

  = (1.645^2^ × 0.50 × 0.50) / 0.10^2^

  = 68

Where,

n = minimum required sample size
Z = 1.645 at 90% Confidence Interval (CI)
p = prevalence taken as 50% for maximum sample size calculation
q = 1-p
e = margin of error, 10%

The calculated sample size is 68, however, total sample size taken was 69. The clinical details were recorded in a predesigned structured proforma which comprised demographic and baseline characteristics, a detailed smoking history, previous treatment history, details of imaging findings, and diagnostic investigations. The patients were investigated by computed tomography (CT) guided fine needle aspiration biopsy, bronchoscopy with transbronchial biopsy, and pleural fluid analysis. The baseline laboratory investigations, and treatment details were also noted. The proforma also collected information on symptoms like fever, hemoptysis, cough, chest pain, loss of appetite, vomiting, weight loss, dyspnea, and orthopnea in the patients. Similarly, the study also focused on looking up the different signs like cyanosis, clubbing, oedema, palpable lymph nodes, and others in the patients. Data were entered and analysed in the Statistical Package for the Social Sciences version 21.0. Point estimate at 90% Confidence Interval and descriptive statistics were interpreted as frequency, percentage, or as mean and standard deviations.

## RESULTS

**Figure 1:**
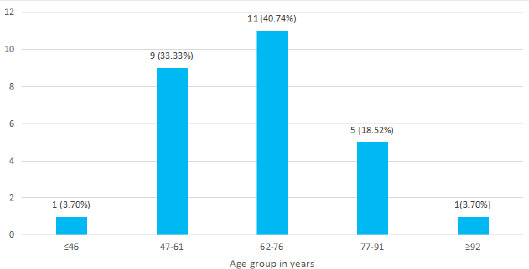
Prevalence of adenocarcinoma age-wise (n= 27).

Out of 69 patients diagnosed with lung cancer, adenocarcinoma was seen in 27 (39.13%) (29.47-48.79 at 90% Confidence Interval). These 69 individuals were aged 43-97 years. The mean age of the patients at the time of diagnosis was 67.59±11.91 years with the majority from the age group 62-76 years (Figure 1).

**Figure 2:**
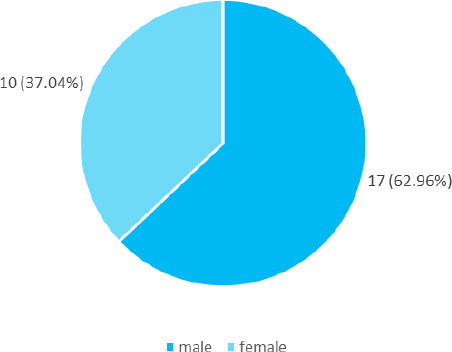
Prevalence of adenocarcinoma gender-wise (n= 27).

Out of 27, 10 (37.04%) were males and 17 (62.96%) were females (Figure 2).

**Figure 3:**
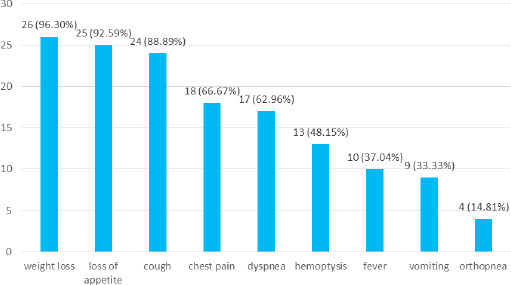
Symptoms at the time of presentation in adenocarcinoma cases (n= 27).

The most common symptom was weight loss, which was seen in 26 (96.30%) patients, followed by loss of appetite in 25 (92.59%), and cough in 24 (88.89%). The other symptoms seen in the patients were chest pain 18 (66.67%), dyspnea 17 (62.86%), hemoptysis 13 (48.15%), fever 10 (37.04%), vomiting 9 (33.33%), and orthopnea 4 (14.81%) (Figure 3).

**Figure 4:**
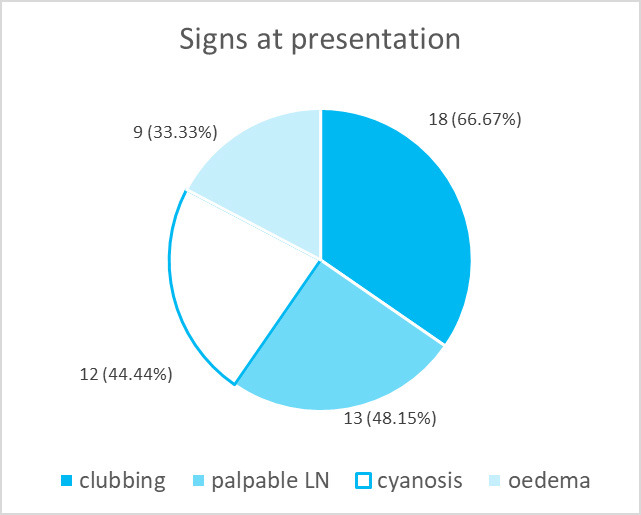
Signs at the time of presentation in adenocarcinoma cases (n= 27).

The most common sign, which was observed among the patients was clubbing in 18 (66.67%) patients followed by palpable lymph nodes in 13 (48.15%), cyanosis in 12 (44.44%), and oedema in 9 (33.33%) (Figure 4).

## DISCUSSION

Adenocarcinoma lung has been the most common type of lung cancer after the decline in the incidence of squamous cell carcinoma since the early 1980s.^5^ In this study, adenocarcinoma was seen in 39.13% of total studied lung cancer cases. A similar finding was observed in an earlier study reported from Nepal which demonstrated adenocarcinoma as the most common lung cancer type with 37.8% prevalence.^6^ There is no population-based National Cancer Registry in Nepal, therefore, there is a lack of data regarding lung cancer incidence in Nepal.

The mean age of patients in this study was 67.59±11.91 years. More than half of new lung neoplasm cases occur in people over 65 years.^7^ Older ages are associated with cancer development due to biological factors that include DNA damage over time and shortening telomeres.^8^ A study represented that adenocarcinoma lung is the most common form of lung cancer in younger women and Asian populations.^4^ Female to male ratio in this study was 1.7:1. However, the age of females was not consistent with the study done.^4^ This may be due to the late presentation of cases in our hospital.

The most common symptom at the time of presentation in this study was weight loss and loss of appetite followed by chronic cough, chest pain, dyspnea, hemoptysis and others. According to the American Cancer Society, symptoms in lung neoplasms in order of higher frequency are cough, hemoptysis, hoarseness, loss of appetite, unexplained weight loss and others.^9^ The most common signs in the present study were clubbing followed by a palpable lymph node, cyanosis and oedema. A similar study carried out in Poland in 2013 reported weight loss, lack of appetite, weakness and fatigue in a significant number of lung cancer patients.^10^

According to one of the study, the risk of adenocarcinoma of the lung increases markedly after a long duration of tobacco smoking.^11^ Since 1985, lung cancer has been the most common cancer caused by smoking.^12^ Though adenocarcinoma is associated with cigarette smoking, it is the most common histology in never smokers.^13^ The proportion of adenocarcinomas is rising in many countries in parallel to an increased incidence of lung cancer in females. These findings may highlight differences in the types of cigarettes including filtered and low-tar versions more frequently used by females as well as genetic predisposition and environmental exposures in female never-smokers.^14^ In our study, more smokers presented with adenocarcinoma than nonsmokers. Specific comorbidities highly prevalent in lung neoplasm patients, include Chronic Obstructive Pulmonary Disease (COPD), hypertension, cardiovascular disease, Diabetes Mellitus (DM) and other malignancies according to review article 2013.^15^

It is a single centre study, hence the findings of this study can not be generalised to the whole population.

## CONCLUSIONS

The prevalence of adenocarcinoma in this study was similar to other studies done in similar settings. Further studies need to be conducted at national level in order to determine the prevalence of adenocarcinoma among lung cancer patients.
